# Energy Transfer in Dy^3+^ and Tb^3+^ Double-Doped Barium Borate Glass

**DOI:** 10.3390/ma16072596

**Published:** 2023-03-24

**Authors:** Michelle Grüne, Stefan Schweizer

**Affiliations:** 1 Faculty of Electrical Engineering, South Westphalia University of Applied Sciences, Lübecker Ring 2, 59494 Soest, Germany; 2 Fraunhofer Application Center for Inorganic Phosphors, Branch Lab of Fraunhofer Institute for Microstructure of Materials and Systems IMWS, Lübecker Ring 2, 59494 Soest, Germany

**Keywords:** borate glass, double doping, energy transfer, light converter, dysprosium, terbium, luminescence, lifetime

## Abstract

In this work, single- and double-doped Dy${}^{3+}$ and Tb${}^{3+}$ barium borate glasses are investigated for their potential as light converters. The density and the absorption coefficient show linearly increasing trends with an increasing lanthanide content. The external quantum efficiency of the double-doped samples is a combination of the respective single-doped samples. The strong energy transfer from Dy${}^{3+}$ to Tb${}^{3+}$ results in an intense Tb${}^{3+}$-related emission, i.e., an intense green luminescence. Thus, excitation at a Dy${}^{3+}$-related wavelength of 452 nm enables a Tb${}^{3+}$-related emission, at which a single-doped Tb${}^{3+}$ sample barely shows any luminescence. Lifetime measurements show that there is not only an energy transfer from Dy${}^{3+}$ to Tb${}^{3+}$, but also vice versa.

## 1. Introduction

To find a suitable light converter at a common laser wavelength in the blue spectral range, a material is needed that can withstand the high thermal load on the one hand and meets the requirements of an efficient light converter on the other. Borate glass offers not only good thermal and chemical stability, but also good solubility for lanthanide ions [1,2,3]. To overcome the luminance limit in the green–yellow spectral range, the so called “green gap” [4,5] of modern light sources, the idea is to use a light converter to convert blue light into the demanded green light. Here, borate glass represents a promising option for this kind of light conversion [6]. As dysprosium (Dy${}^{3+}$) and terbium (Tb${}^{3+}$) enable an intense green–yellowish (Dy${}^{3+}$) to green (Tb${}^{3+}$) luminescence, they are interesting candidates for the double doping of borate glass. In this work, Dy${}^{3+}$ and Tb${}^{3+}$ single- and double-doped barium borate (BaB) glasses are investigated for their luminescence properties, in particular for excitation in the blue spectral range.

## 2. Materials and Methods

### 2.1. Samples

To prepare the samples under study, boron oxide (B${}_{2}$O${}_{3}$ from Alfa Aesar, 99% purity) is used as a network former and barium oxide (BaO from Sigma-Aldrich, 97% purity) is used as a network modifier. The ratio of network modifier to network former is 1:4. To optically activate the glasses, the lanthanide oxides, dysprosium oxide (Dy${}_{2}$O${}_{3}$ from Alfa Aesar, 99.99% purity) and terbium oxide (Tb${}_{4}$O${}_{7}$ from Alfa Aesar, 99.9% purity), are used. Both lanthanide oxides are added at the expense of the network former and network modifier content. The nominal chemical composition of the investigated glasses are listed in Table 1.

**Figure 1:**
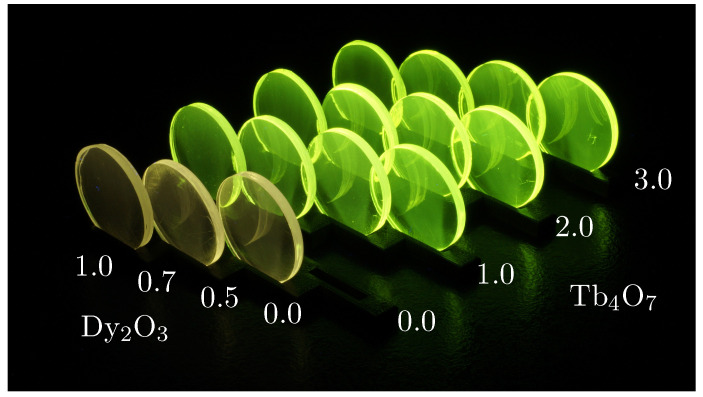
Sample series of Dy${}^{3+}$ and Tb${}^{3+}$ single- and double-doped BaB glasses with different Dy${}^{3+}$ and Tb${}^{3+}$ contents under 365 nm excitation. From left to right, the Dy${}_{2}$O${}_{3}$ content is 1.0, 0.7, 0.5, and 0.0; from front to back, the Tb${}_{4}$O${}_{7}$ content amounts to 0.0, 1.0, 2.0, and 3.0 (all values are in mol%).

For the preparation of the samples, the chemicals are mixed and melted in a platinum gold crucible (Pt/Au 95/5) for 3 hours at 1100 ${}^{\circ }C$. Subsequently, the melt is poured onto a 550 ${}^{\circ }C$ pre-heated brass block, which is below the glass transition temperature of BaB glass ($T_{g}=605\;{}^{\circ }C$) [7]. To eliminate residual internal stress, the glass is held at this temperature for 3 hours before being cooled down to room temperature in a controlled manner. The samples are produced in a circular shape with a diameter of 25.4 mm (1 inch). They are ground to a thickness of 2.6 mm and subsequently polished to optical quality from both sides (Figure 1).

### 2.2. Setup

The mass density of each glass sample is measured by the Archimedes’ principle with a density determination kit for an analytical balance (Mettler Toledo XS105DU). For each value, ten measurements are performed, the arithmetic mean is listed in Table 1. The experimental error amounts to $\pm 0.005\;g/cm^{3}$.

Transmittance measurements are performed with a UV-Vis-NIR spectrophotometer (Agilent Technologies Cary 5000). Photoluminescence emission spectra as well as external quantum efficiency are measured with an absolute photoluminescence quantum yield measurement system (Hamamatsu C9920-02G). This system comprises a xenon lamp ( 150 W) for excitation, which is connected via a monochromator to a 3.3-inch integrating sphere, and a photonic multi-channel analyzer (Hamamatsu PMA-12) for detection.

For photoluminescence lifetime measurements, a 375-nm laser diode (Nichia NDU4116) and a 455-nm laser diode (Laser Components FP-D-450-40D-C-F) are used for excitation. The photoluminescence emission is detected with a Peltier-cooled photomultiplier (Hamamatsu R943-02) coupled to a 300-mm focal length monochromator (Princeton Instruments Acton 2300). The laser diode is switched on and off with a 20-Hz square wave function (Rhode & Schwarz Arbitrary Function Generator HMF 2550). The photomultiplier signal is recorded with a digital storage oscilloscope (Rhode & Schwarz HMO 1024) and a 10-kΩ resistor in parallel.

## 3. Results

### 3.1. Mass Density

**Figure 2:**
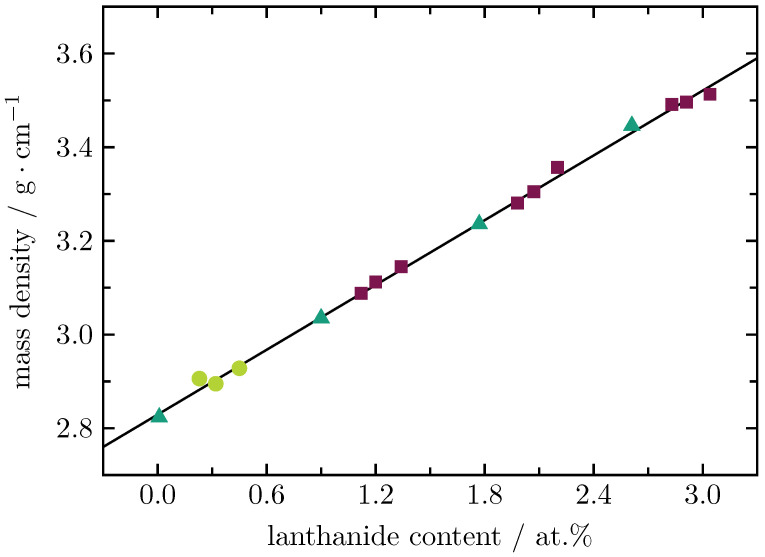
Mass densities of Dy${}^{3+}$ (full circles) and Tb${}^{3+}$ (full triangles) single-doped as well as Dy${}^{3+}$/Tb${}^{3+}$ (full squares) double-doped BaB glasses. The solid line represents a least squares fitting to the experimental data.

The measured mass densities show a linear increase upon increasing the lanthanide content (Figure 2). Note, that there is no significant difference between the molar mass of Dy${}^{3+}$ (162.50 g/mol) and that of Tb${}^{3+}$ (158.93 g/mol). This is in very good agreement with the findings for Dy${}^{3+}$ single-doped LiAlB glass [8], where the mass density also increases linearly with an increasing Dy${}_{2}$O${}_{3}$ concentration.

### 3.2. Absorption and Photoluminescence Quantum Efficiency

**Figure 3:**
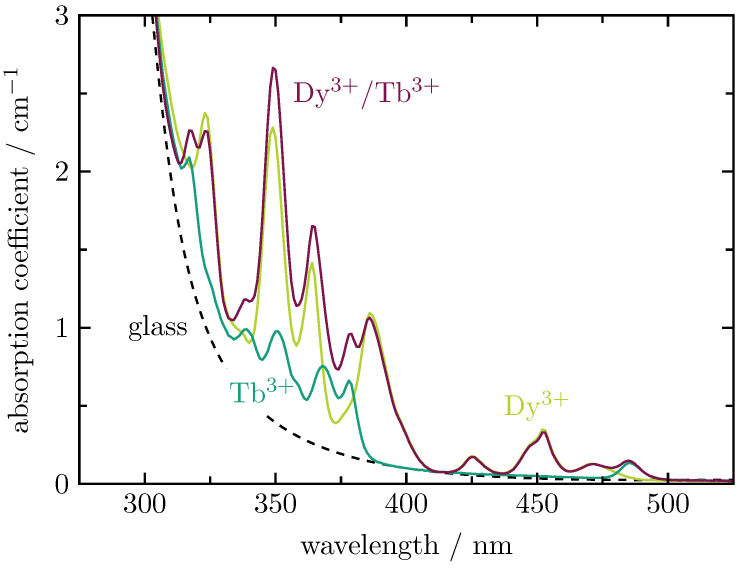
Absorption coefficients of Dy${}^{3+}$ (0.45 at.%) and Tb${}^{3+}$ (0.90 at.%) single-doped as well as Dy${}^{3+}$/Tb${}^{3+}$ (0.45 at.%/0.89 at.%) double-doped BaB glasses. The glass background is indicated by the dashed line.

**Figure 4:**
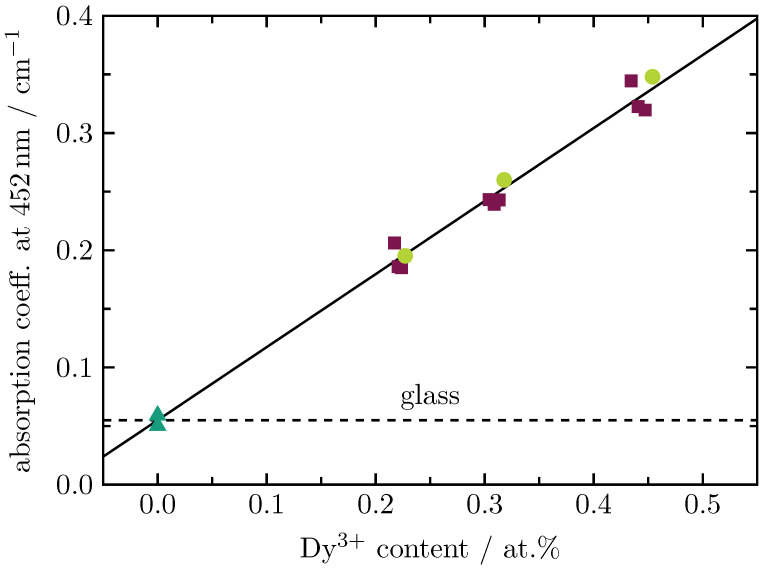
Total absorption coefficient at 452 nm versus Dy${}^{3+}$ content for Dy${}^{3+}$ (full circles) and Tb${}^{3+}$ (full triangles) single-doped as well as Dy${}^{3+}$/Tb${}^{3+}$ (full squares) double-doped BaB glasses. The solid line represents a least squares fitting to the experimental data. The glass background is indicated by the dashed line.

The absorption coefficients, shown exemplarily in Figure 3 for Dy${}^{3+}$ (0.45 at.%) and Tb${}^{3+}$ (0.90 at.%) single-doped glasses as well as the corresponding Dy${}^{3+}$/Tb${}^{3+}$ double-doped BaB glass, are obtained from transmission measurements (not shown here). The spectra show clearly how the absorption bands of Dy${}^{3+}$ (light green curve) and Tb${}^{3+}$ (dark green curve) sum up for the Dy${}^{3+}$/Tb${}^{3+}$ double-doped sample (wine-red curve). Note that the absorption coefficient still includes the glass background (dashed curve), which starts at approximately 500 nm. The total absorption coefficients at a wavelength of 452 nm are displayed in Table 2. Figure 4 shows a linear dependence of the total absorption coefficient at 452 nm on the Dy${}^{3+}$ content. The dashed line indicates the contribution of the glass background to the total absorption at this wavelength.

**Figure 5:**
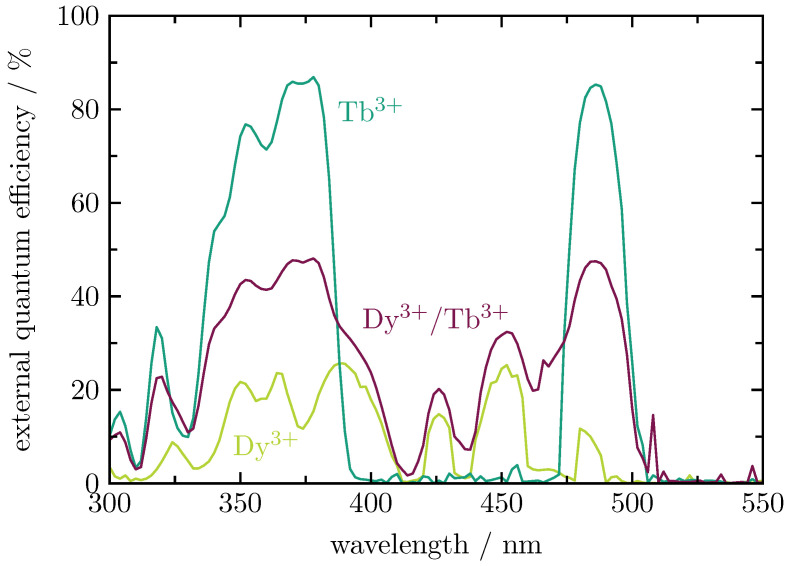
External quantum efficiencies of Dy${}^{3+}$ (0.23 at.%) and Tb${}^{3+}$ (2.61 at.%) single-doped as well as Dy${}^{3+}$/Tb${}^{3+}$ (0.22 at.%/2.61 at.%) double-doped BaB glasses.

The external quantum efficiency spectra (Figure 5) show that, for Tb${}^{3+}$, the excitation wavelength of interest is at 378 nm, where the ${}^{7}$F${}_{6}$ → ${}^{5}$D${}_{3}$ transition occurs. For Dy${}^{3+}$, the interesting wavelengths are 386 nm and 452 nm due to the ${}^{6}$H${}_{15/2}$→${}^{4}$I${}_{13/2}$ and the ${}^{6}$H${}_{15/2}$→${}^{4}$I${}_{15/2}$ transitions, respectively [9]. At these wavelengths, the quantum efficiency spectra of the single-doped glasses show their maxima. At 378 nm, Tb${}^{3+}$ reaches a quantum efficiency of 86%, while Dy${}^{3+}$ has a maximum quantum efficiency of 25% at 386 nm as well as at 452 nm. The combination of the two lanthanides in the double-doped glass (0.22 at.%/2.61 at.% Tb${}^{3+}$) shows a quantum efficiency of 48% at 378 nm, 36% at 386 nm, and 32% at 452 nm.

### 3.3. Photoluminescence and Energy Transfer

**Figure 6:**
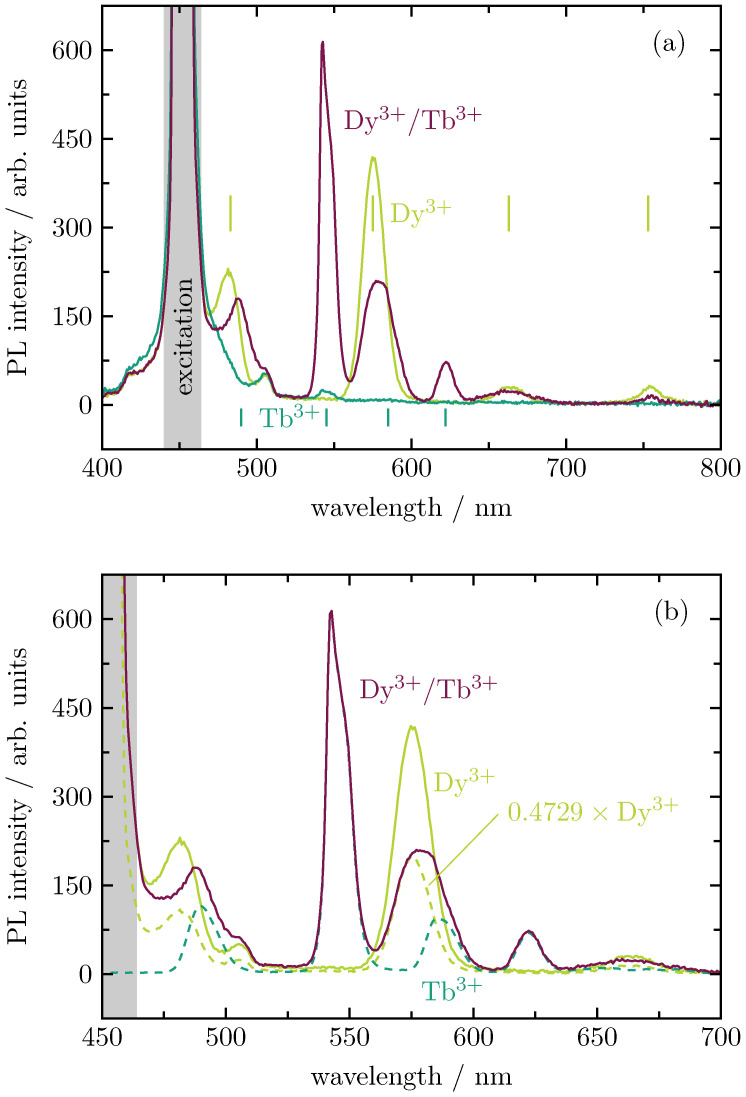
(**a**) Photoluminescence (PL) emission spectra of Dy${}^{3+}$ (0.45 at.%) and Tb${}^{3+}$ (0.90 at.%) single-doped as well as Dy${}^{3+}$/Tb${}^{3+}$ (0.45 at.%/0.89 at.%) double-doped BaB glasses; the emission spectra are recorded under 452-nm excitation. (**b**) Deconvolution of the emission spectrum of the Dy${}^{3+}$/Tb${}^{3+}$ double-doped BaB glass.

As 452 nm is a common laser wavelength, the behavior of the double-doped samples under blue excitation is investigated. The photoluminescence emission spectra of the Dy${}^{3+}$ and Tb${}^{3+}$ single-doped samples as well as the Dy${}^{3+}$ / Tb${}^{3+}$ double-doped sample are shown in Figure 6a. The Dy${}^{3+}$ doped glass (light green curve) shows four maxima at 483 nm, 575 nm, 663 nm, and 753 nm, which belong to transitions from the excited state ${}^{4}$F${}_{9/2}$ to the ground states ${}^{6}$H${}_{15/2}$, ${}^{6}$H${}_{13/2}$, ${}^{6}$H${}_{11/2}$, and ${}^{6}$H${}_{9/2}$, respectively [9]. The mixture of the largest emission band at 575 nm and the second largest emission band at 483 nm results in the green–yellowish color impression of Dy${}^{3+}$. The Tb${}^{3+}$ doped glass (dark green curve) also shows four maxima, namely at 490 nm, 542 nm, 585 nm, and 622 nm due to transitions from the excited state ${}^{5}$D${}_{4}$ to the ground states ${}^{7}$F${}_{6}$, ${}^{7}$F${}_{5}$, ${}^{7}$F${}_{4}$, and ${}^{7}$F${}_{3}$, respectively [9]. By far the largest emission band is the one at 542 nm, which is also responsible for the green color impression of Tb${}^{3+}$.

Due to the energy transfer from Dy${}^{3+}$ to Tb${}^{3+}$ [10], the double-doped glass (wine-red curve) shows almost the same emission spectrum as the Tb${}^{3+}$ single-doped glass. However, as Tb${}^{3+}$ cannot be excited well at 452 nm, but Dy${}^{3+}$ can, the photoluminescence emission spectrum of the double-doped sample is significantly higher than that of the single-doped Tb${}^{3+}$ sample. It becomes clear that at the Dy${}^{3+}$-related excitation wavelengths, the double-doped glass shows the best performance. The Tb${}^{3+}$ single-doped glass shows almost no emission upon excitation at 452 nm. The Dy${}^{3+}$ single-doped sample emits well at these excitation wavelengths, but the result is not as intense as the double-doped glass, whose Tb${}^{3+}$-related emission intensity is more than doubled.

Figure 6b shows how the emission spectrum of the double-doped glass (solid wine-red curve, excited at 452 nm) is composed of the emission spectra of the single-doped glasses. The solid light green curve shows the emission spectrum of the single-doped Dy${}^{3+}$ glass, excited at 452 nm. The two dashed curves show the emission spectrum of the Dy${}^{3+}$ single-doped glass (light green, excited at 452 nm) and the Tb${}^{3+}$ single-doped glass (dark green, excited at 378 nm) with the ratio they contributed to the emission spectrum of the double-doped glass.

**Figure 7:**
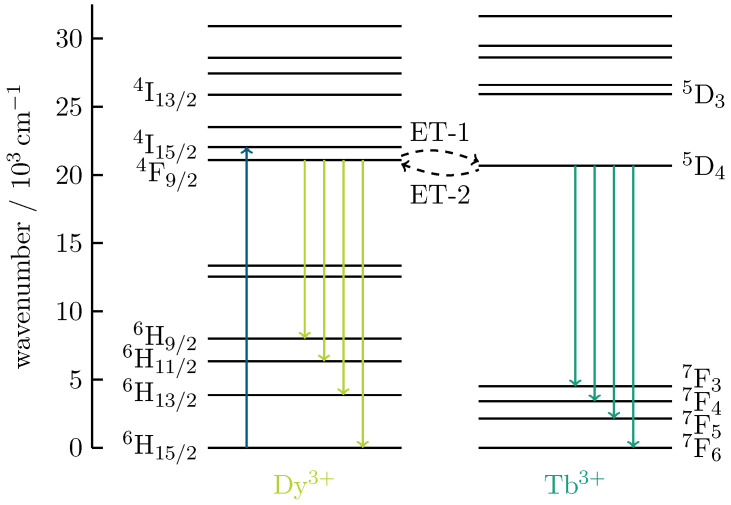
Energy level diagrams depicting the 452-nm excitation route of Dy${}^{3+}$ (blue arrow) and the routes for radiative emissions from the ${}^{4}$F${}_{9/2}$ level of Dy${}^{3+}$ and the ${}^{5}$D${}_{4}$ level of Tb${}^{3+}$. The dashed arrows indicate the energy transfer from Dy${}^{3+}$ to Tb${}^{3+}$, and vice versa.

The condition for energy transfer is when the states of the acceptor (Tb${}^{3+}$) are the same or only slightly different from the excited states of the donor (Dy${}^{3+}$). Thus, resonant energy transfer of the excitation energy of the donor results in re-absorption by the acceptor. This occupies an excited level, which entails the possibility of a visible emission [11,12,13]. For an excitation wavelength of 452 nm, Dy${}^{3+}$ emits a green–yellowish color spectrum, as shown in Figure 6a. As the excited state of Dy${}^{3+}$ is almost on the same level as that of Tb${}^{3+}$, energy transfer from Dy${}^{3+}$ to Tb${}^{3+}$, and vice versa, can occur here. The most probable channel for the energy transfer is through non-radiative relaxation corresponding to the Dy${}^{3+}$-related ${}^{4}$F${}_{9/2}$→${}^{6}$H${}_{15/2}$ transition and Tb${}^{3+}$ excitation from ${}^{7}$F${}_{6}$ to ${}^{5}$D${}_{4}$, as illustrated in Figure 7.

**Figure 8:**
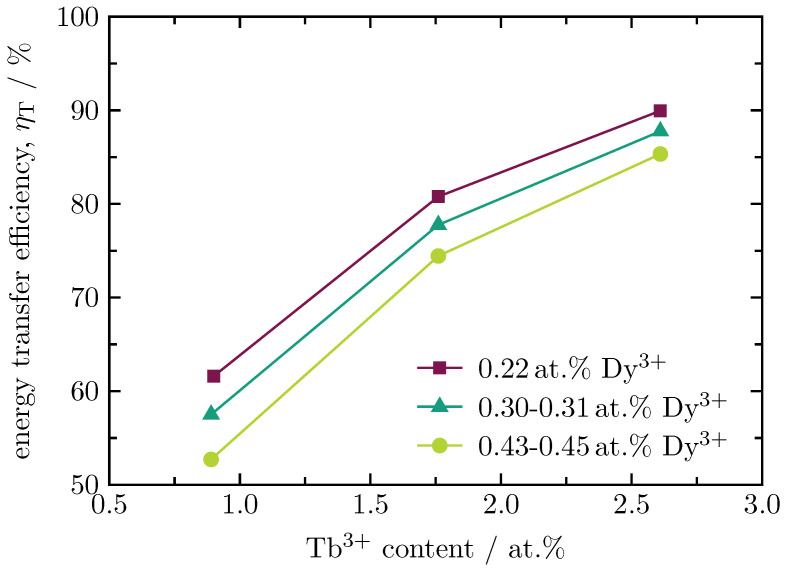
Energy transfer efficiency from Dy${}^{3+}$ to Tb${}^{3+}$ ions for varying Dy${}^{3+}$ contents as a function of the Tb${}^{3+}$ content.

Compared to other glass systems [14,15,16,17,18,19,20,21,22], the energy transfer is of special interest here. As can be seen in Figure 6a, Tb${}^{3+}$ shows almost no emission when excited with a Dy${}^{3+}$-related excitation wavelength, i.e., 452 nm. The double-doped samples, however, show a strong Tb${}^{3+}$-related emission. To calculate the efficiency of the dipole–dipole energy transfer, Förster’s model [23,24] is used, which calculates the energy transfer efficiency based on a change in fluorescence intensity in the presence and absence of the acceptor,

(1) $$\begin{array}{c} \eta_{T}=1-\frac{I}{I_{0}}, \end{array}$$

where $\eta_{T}$ is the energy transfer efficiency, *I* is the fluorescence intensity at the maximum emission of the donor in presence of the acceptor, and $I_{0}$ is the fluorescence intensity at the maximum emission of the donor in absence of the acceptor. To determine the energy transfer, the intensity of the main peak of the donor at 575 nm of a Dy${}^{3+}$ single-doped sample is detected first. Subsequently, the intensities of the double-doped samples at 575 nm are detected. For the calculation of the energy transfer, the values are inserted into Equation (1). Figure 8 shows the energy transfer efficiency of the sample series as a function of the Tb${}^{3+}$ content. It can be seen that the highest energy transfer efficiency is achieved for the samples with 0.22 at.% Dy${}^{3+}$. The decrease in efficiency at higher Dy${}^{3+}$ doping levels can be explained by the quenching effect [8,25]. In contrast to this, the energy transfer efficiency grows with increasing Tb${}^{3+}$ content, as the interatomic distance between Dy${}^{3+}$ and Tb${}^{3+}$ decreases. The highest value is obtained for the sample containing 2.61 at.% Tb${}^{3+}$. All resulting energy transfer efficiencies are listed in Table 2.

### 3.4. Photoluminescence Lifetime and Energy Transfer

**Figure 9:**
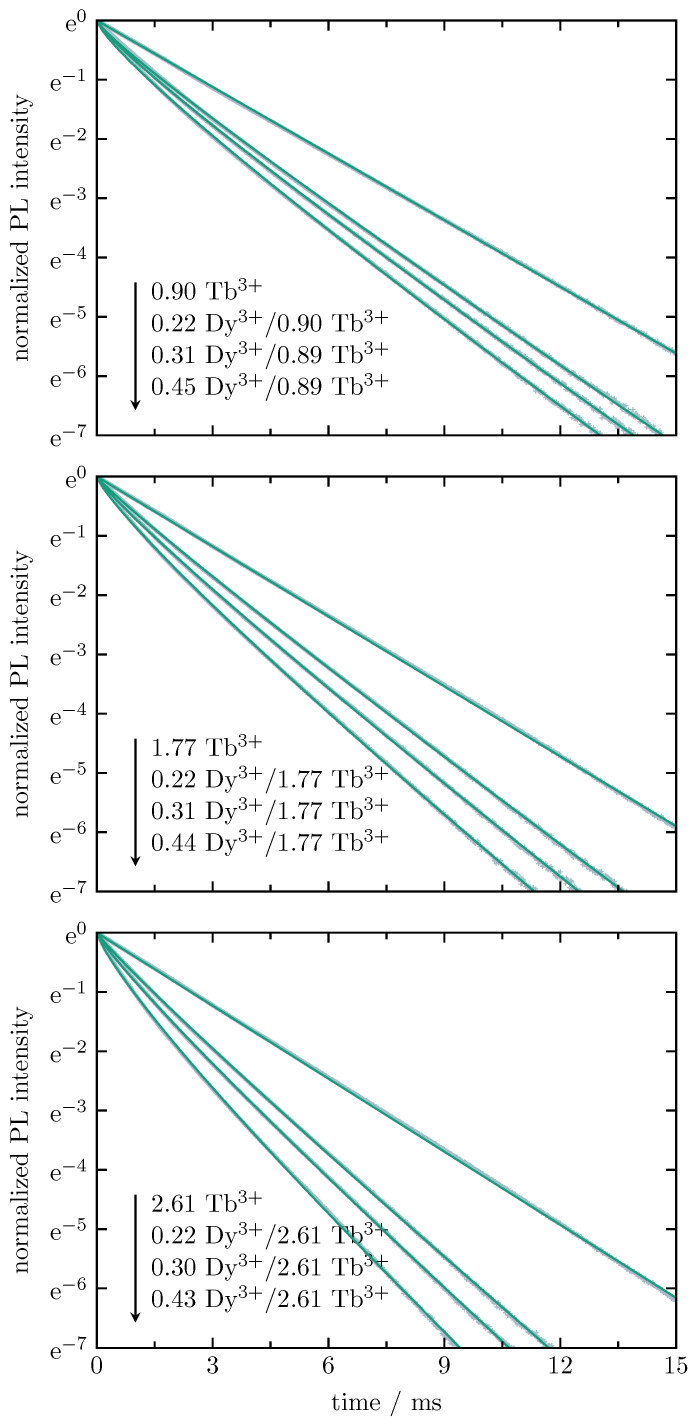
Normalized radiative decay curves of Tb${}^{3+}$ in Tb${}^{3+}$ for single- as well as Dy${}^{3+}$/Tb${}^{3+}$ double-doped BaB glasses with varying Tb${}^{3+}$ and Dy${}^{3+}$ contents (all values are in at.%), recorded for the Tb${}^{3+}$-related ${}^{5}$D${}_{4}$ to ${}^{7}$F${}_{5}$ transition at 545 nm under 375-nm (Tb${}^{3+}$ single-doped glasses) and 455-nm (Dy${}^{3+}$/Tb${}^{3+}$ double-doped glasses) excitations. The solid curves (green) represent the best fit on the basis of the Inokuti–Hirayama model.

Figure 9 shows the normalized radiative decay curves of Tb${}^{3+}$ in Tb${}^{3+}$ single-doped as well as in Dy${}^{3+}$/Tb${}^{3+}$ double-doped BaB glasses with varying Tb${}^{3+}$ and Dy${}^{3+}$ contents. The decay is recorded for the Tb${}^{3+}$-related emission at 545 nm (${}^{5}$D${}_{4}$ to ${}^{7}$F${}_{5}$ transition) under 375-nm (Tb${}^{3+}$ single-doped glasses) and 455-nm excitation (Dy${}^{3+}$/Tb${}^{3+}$ double-doped glasses), i.e., under excitation of the Tb${}^{3+}$-related ${}^{7}$F${}_{6}$ to ${}^{5}$D${}_{3}$ and the Dy${}^{3+}$-related ${}^{6}$H${}_{15/2}$ to ${}^{4}$I${}_{15/2}$ transitions, respectively. The decay curves for the Tb${}^{3+}$ single-doped glasses follow a mono-exponential behavior, whereas the Tb${}^{3+}$ decay in the double-doped glasses do not. In the latter case, the relaxation comprised radiative and non-radiative processes. Non-radiative decays include Inokuti-phonon relaxation and ion–ion interactions, such as energy transfer and cross-relaxation.

For the Tb${}^{3+}$ decay, the non-radiative energy transfer occurs between neighbouring Tb${}^{3+}$ and Dy${}^{3+}$ ions arising from multipolar interaction. The Inokuti–Hirayama model [26] is an attempt to describe this behavior:

(2) $$\begin{array}{c} I\left(t\right)=I_{0}\;\cdot \;\exp\left(-\frac{t}{\tau_{0}}-\gamma \;\cdot \;{\left(\frac{t}{\tau_{0}}\right)}^{3/S}\right) \end{array}$$

where I(t) is the intensity of the radiative decay, $I_{0}$ is the initial intensity, *t* is the time after the excitation pulse, $\tau_{0}$ is the intrinsic lifetime of the donor in the absence of the acceptor, γ is the energy transfer parameter, and *S* is the multipolar interaction parameter. The value *S* corresponds to electrical dipole–dipole, dipole–quadrupole, and quadrupole–quadrupole interactions when equal to 6, 8, and 10, respectively. In this case, Tb${}^{3+}$ is the donor and Dy${}^{3+}$ is the acceptor. The results obtained from a best fit on the basis of the above-described Inokuti–Hirayama model are shown as green solid curves in Figure 9; the corresponding fitting parameters are listed in Table 3. For each Tb${}^{3+}$ series, the observed experimental lifetime, $\tau_{\exp}$, decreases with an increasing Dy${}^{3+}$ content, whereas the energy transfer parameter, γ, increases.

The intrinsic lifetime, $\tau_{0}$, of Tb${}^{3+}$ in single-doped BaB glass ranges from 2.86 ms (0.01 at.%) to 2.44 ms (2.61 at.%). The shorter lifetime for increasing Tb${}^{3+}$ content is caused by the Tb${}^{3+}$–Tb${}^{3+}$ interaction. These values are very similar to those found for other borate glasses. In Loos et al. [27], an intrinsic lifetime of 2.5 ms is found for a BaB glass with a Tb${}^{3+}$ content of 1.0 at.%, while the network modifier to network former ratio is 1:2. Padlyak and Drzewiecki [28] investigated Tb${}^{3+}$ single-doped CaB${}_{4}$O${}_{7}$ and LiCaBO${}_{3}$ glasses containing 0.5 mol% and 1.0 mol% Tb${}_{2}$O${}_{3}$. For both glasses, they found a mono-exponential decay with Tb${}^{3+}$ lifetimes of 2.43 ms (0.5 mol%) and 2.30 ms (1.0 mol%) for CaB${}_{4}$O${}_{7}$ glass and 2.40 ms (0.5 mol%) and 2.35 ms (1.0 mol%) for LiCaBO${}_{3}$ glass. The authors claim that the slight difference between the obtained values is caused by the glass host structure. For glasses with the same Tb${}^{3+}$ content, the lifetime decreases with a decreasing distance of Tb${}^{3+}$ to the surrounding oxygen ions. In both systems, Tb${}^{3+}$ is localised in a Li/Ca site, coordinated by O${}^{2-}$ ions (coordination number N=4 to 7 with statistically distributed Tb${}^{3+}$-O${}^{2-}$ distances). For CaB${}_{4}$O${}_{7}$ glass, the interatomic distance amounts to 0.262 nm, while it is 0.258 nm in the case of LiCaBO${}_{3}$ glass.

The fitted values of the multipolar interaction parameter, *S*, are all smaller, but close to 6, indicating that electrical dipole–dipole interaction is mainly responsible for the energy transfer from Tb${}^{3+}$ to Dy${}^{3+}$. In addition to the energy transfer from Dy${}^{3+}$ to Tb${}^{3+}$, as analyzed above by deconvolution of the corresponding emission spectra, there is also an energy transfer from Tb${}^{3+}$ to Dy${}^{3+}$.

This efficiency can be determined from the lifetime measurements. Based on Förster’s model [23], the energy transfer efficiency is given by

(3) $$\begin{array}{c} \eta_{T}=1-\frac{\tau_{\exp}}{\tau_{0}}, \end{array}$$

where the energy transfer efficiency is $\eta_{T}$, the experimental lifetime is $\tau_{\exp}$, and the intrinsic lifetime is $\tau_{0}$. The results determined this way are collected in the last column of Table 3. As already observed for the energy transfer from Dy${}^{3+}$ to Tb${}^{3+}$, the best results are obtained for the samples with 2.61 at.% Tb${}^{3+}$, the values increase with an increasing Dy${}^{3+}$ concentration. The highest energy transfer efficiency of 61% is obtained for the sample with 0.43 at.% Dy${}^{3+}$ and 2.61 at.% Tb${}^{3+}$, i.e., the highest amount of Dy${}^{3+}$ as well as Tb${}^{3+}$.

**Figure 10:**
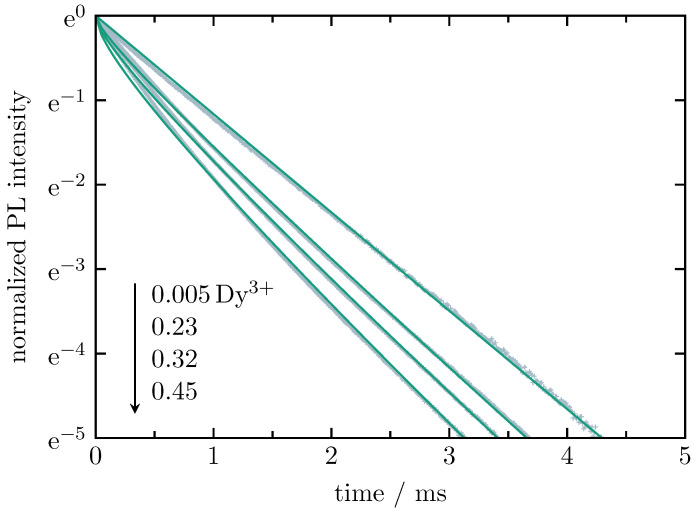
Normalized radiative decay curves of Dy${}^{3+}$ in Dy${}^{3+}$ single-doped BaB glass with varying Dy${}^{3+}$ contents (all values are in at.%), recorded for the Dy${}^{3+}$-related ${}^{4}$F${}_{9/2}$ to ${}^{6}$H${}_{13/2}$ transition at 575 nm under 455-nm excitation. The solid curves (green) represent the best fit on the basis of the Inokuti–Hirayama model with the multipolar interaction parameter, *S*, set to 6 (dipole–dipole interaction).

The decay of Dy${}^{3+}$ in Dy${}^{3+}$ single-doped samples is shown in Figure 10. The analysis is also performed on the basis of the Inokuti–Hirayama model with the multipolar interaction parameter, *S*, set to 6 (dipole–dipole interaction) to allow for a direct comparison with previous lifetime measurements [25]. The decay of the 0.005 at.%-doped sample is assumed to be mono-exponential. The resulting decay time serves as intrinsic decay time, $\tau_{0}$, for the fit of the decay curves of the 0.23 at.%-, 0.32 at.%-, and 0.45 at.%-doped samples. The fit parameters are close to those found in previous measurements [25]. The experimental lifetime, $\tau_{\exp}$, decreases from 0.86 ms (0.005 at.%) to 0.47 ms (0.45 at.%) with an increasing Dy${}^{3+}$ content, whereas the energy transfer parameter, γ, increases from 0.359 (0.23 at.%) to 0.712 (0.45 at.%). The fitted intrinsic lifetime, $\tau_{0}$, is approximately 0.9 ms, i.e., it is similar to the previously found value of 917 μs.

## 4. Conclusions

In conclusion, the Dy${}^{3+}$/Tb${}^{3+}$ double-doped BaB glass series shows a linear growth with an increasing lanthanide content for the mass density as well as for the absorption coefficient at 452 nm. At this wavelength, the external quantum efficiency achieves a maximum value of 35% for the sample with 0.22 at.% Dy${}^{3+}$ and 1.76 at.% Tb${}^{3+}$. Deconvolution of the emission spectra yields a value for the energy transfer efficiency of 90% for the sample with 0.22 at.% Dy${}^{3+}$ and 2.61 at.% Tb${}^{3+}$. Though Tb${}^{3+}$ can not be excited at a wavelength of 452 nm, a significant Tb${}^{3+}$-related luminescence is obtained for this wavelength due to the strong energy transfer from Dy${}^{3+}$ to Tb${}^{3+}$. Lifetime measurements show that there is also an energy transfer from Tb${}^{3+}$ to Dy${}^{3+}$, which is, however, not as intense as the energy transfer from Dy${}^{3+}$ to Tb${}^{3+}$. As the Dy${}^{3+}$/Tb${}^{3+}$ double-doped glass shows an intense green luminescence under excitation in the blue spectral range, this system has a huge potential to overcome the green-gap problem.

## Figures and Tables

**Table 1:** Nominal composition, lanthanide content, mass density, molar mass, and molar volume of the BaB glasses investigated. The experimental error of the density values amounts to $\pm 0.005\;g/cm^{3}$.

Composition in mol%				La${}^{3+}$ Content in at.%		Mass Density	Molar Mass	Molar Volume
**B** ${}_{2}$ **O** ${}_{3}$	**BaO**	**Dy** ${}_{2}$ **O** ${}_{3}$	**Tb** ${}_{4}$ **O** ${}_{7}$	**Dy** ${}^{3+}$	**Tb** ${}^{3+}$	**In g/cm** ${}^{3}$	**In g/mol**	**In cm** ${}^{3}$
79.60	19.90	0.5	–	0.23	–	2.906	187.79	30.21
78.80	19.70	0.5	1.0	0.22	0.90	3.088	194.41	30.57
78.00	19.50	0.5	2.0	0.22	1.76	3.281	101.02	30.79
77.20	19.30	0.5	3.0	0.22	2.61	3.491	107.63	30.83
79.44	19.86	0.7	–	0.32	–	2.895	188.37	30.53
78.64	19.66	0.7	1.0	0.31	0.89	3.112	194.98	30.52
77.84	19.46	0.7	2.0	0.31	1.76	3.305	101.59	30.74
77.04	19.26	0.7	3.0	0.30	2.61	3.496	108.21	30.96
79.20	19.80	1.0	–	0.45	–	2.928	189.23	30.47
78.40	19.60	1.0	1.0	0.45	0.89	3.145	195.84	30.47
77.60	19.40	1.0	2.0	0.44	1.76	3.357	102.45	30.52
76.80	19.20	1.0	3.0	0.43	2.61	3.513	109.07	31.04
79.99	20.00	–	0.01	–	0.009	2.824	186.43	30.61
79.20	19.80	–	1.0	–	0.90	3.035	192.97	30.63
78.40	19.60	–	2.0	–	1.77	3.236	199.59	30.77
77.60	19.40	–	3.0	–	2.61	3.446	106.20	30.82

**Table 2:** Concentration of lanthanide ions, $c_{{\mathrm{La}}^{3+}}$; mean Dy${}^{3+}$-to-Tb${}^{3+}$ distance, $d_{{\mathrm{Dy}}^{3+}-{\mathrm{Tb}}^{3+}}$; total absorption coefficients (including the glass background); and external quantum efficiencies of Dy${}^{3+}$ and Tb${}^{3+}$ single-doped as well as Dy${}^{3+}$/Tb${}^{3+}$ double-doped BaB glasses at a wavelength of 452 nm. For the double-doped glasses, the ratios $I/I_{0}$ as well as the resulting energy transfer efficiencies, $\eta_{T}$, are given.

Dy ${}^{3+}$	Tb${}^{3+}$	$c_{{\mathrm{La}}^{3+}}$	$d_{{\mathrm{Dy}}^{3+}-{\mathrm{Tb}}^{3+}}$	$\alpha_{\mathrm{total}}$	EQE	$I/I_{0}$	$\eta_{T}$
**In at.%**	**In at.%**	**In 10** ${}^{20}$ **cm** ${}^{-3}$	**In nm**	**In cm** ${}^{-1}$	**In %**		**In %**
0.23	–			0.195	28	–	–
0.22	0.90	15.91	1.19	0.185	30	0.3840	62
0.22	1.76	19.78	1.01	0.186	35	0.1922	81
0.22	2.61	13.67	0.90	0.206	32	0.1005	90
0.32	–			0.260	21	–	–
0.31	0.89	16.71	1.14	0.243	25	0.4249	58
0.31	1.76	10.58	0.98	0.239	28	0.2224	78
0.30	2.61	14.40	0.89	0.243	26	0.1224	88
0.45	–			0.348	13	–	–
0.45	0.89	17.90	1.08	0.320	18	0.4729	53
0.44	1.76	11.84	0.95	0.323	20	0.2556	74
0.43	2.61	15.52	0.86	0.345	17	0.1467	85
–	0.90			0.059	2	–	–
–	1.77			0.050	3	–	–
–	2.61			0.059	4	–	–

**Table 3:** Fit parameters obtained from an analysis based on the Inokuti–Hirayama model as well as the energy transfer efficiency calculated from the lifetime.

Exc./	Dy${}_{2}$O${}_{3}$	Tb${}_{4}$O${}_{7}$	Dy${}^{3+}$	Tb${}^{3+}$	$\tau_{\exp}$	$\tau_{0}$	γ	*S*	$\eta_{T}$
**Det.**	**In mol%**	**In mol%**	**In at.%**	**In at.%**	**In ms**	**In ms**			**In %**
	–	0.01	–	0.01	2.86				
375 nm/	–	1.0	–	0.90	2.67				
545 nm	–	2.0	–	1.77	2.54				
	–	3.0	–	2.61	2.44				
	0.5	1.0	0.22	0.90	1.73		0.491	4.55	35
	0.7	1.0	0.31	0.89	1.57	2.67	0.598	4.50	41
	1.0	1.0	0.45	0.89	1.37		0.765	4.67	49
455 nm/	0.5	2.0	0.22	1.76	1.70		0.447	3.88	33
545 nm	0.7	2.0	0.31	1.76	1.47	2.54	0.640	4.04	42
	1.0	2.0	0.44	1.76	1.22		0.884	4.24	52
	0.5	3.0	0.22	2.61	1.45		0.610	3.68	41
	0.7	3.0	0.30	2.61	1.25	2.44	0.834	3.88	49
	1.0	3.0	0.43	2.61	0.96		1.195	4.20	61
	0.01	–	0.005	–	0.86				
455 nm/	0.5	–	0.23	–	0.61		0.359	6	
575 nm	0.7	–	0.32	–	0.55	0.86	0.516	6	
	1.0	–	0.45	–	0.47		0.712	6	
